# Rules from Words: A Dynamic Neural Basis for a Lawful Linguistic Process

**DOI:** 10.1371/journal.pone.0086212

**Published:** 2014-01-21

**Authors:** David W. Gow, A. Conrad Nied

**Affiliations:** 1 Neuropsychology Laboratory, Massachusetts General Hospital, Boston, Massachusetts, United States of America; 2 Department of Psychology, Salem State University, Salem, Massachusetts, United States of America; 3 Athinoula A. Martinos Center for Biomedical Imaging, Massachusetts General Hospital, Charlestown, Massachusetts, United States of America; 4 Harvard-MIT Division of Health Sciences and Technology, Cambridge, Massachusetts, United States of America; UNLV, United States of America

## Abstract

Listeners show a reliable bias towards interpreting speech sounds in a way that conforms to linguistic restrictions (phonotactic constraints) on the permissible patterning of speech sounds in a language. This perceptual bias may enforce and strengthen the systematicity that is the hallmark of phonological representation. Using Granger causality analysis of magnetic resonance imaging (MRI)- constrained magnetoencephalography (MEG) and electroencephalography (EEG) data, we tested the differential predictions of rule-based, frequency–based, and top-down lexical influence-driven explanations of processes that produce phonotactic biases in phoneme categorization. Consistent with the top-down lexical influence account, brain regions associated with the representation of words had a stronger influence on acoustic-phonetic regions in trials that led to the identification of phonotactically legal (versus illegal) word-initial consonant clusters. Regions associated with the application of linguistic rules had no such effect. Similarly, high frequency phoneme clusters failed to produce stronger feedforward influences by acoustic-phonetic regions on areas associated with higher linguistic representation. These results suggest that top-down lexical influences contribute to the systematicity of phonological representation.

## Introduction

Language is strikingly systematic and generative. We see its systematicity in the lawful patterning of structure at all levels of linguistic representation, and its generativity in the continuous creation of new forms that observe these regularities. In phonology, the lawful patterning of speech sounds to form syllables and words is described by systematic prohibitions on the sequencing of phonemes termed phonotactic constraints. These constraints inform the intuition that *doke* could be an English word, but *lteg* could not [1]. These principles constrain the creation of new wordforms and the systematic restructuring of loan words [2].

These principles also lead to systematic perceptual biases in nonword perception. Behavioral results show that listeners readily “repair” phonotactic violations either through perceptual shifts in the categorization of phonemes (e.g. hearing *tl-* as/tr −/) or by inserting illusory epenthetic vowels (hearing *tl-*as/t∂l−/) [3], [4], [5], [6]. Recent simulation results [7] demonstrate that regularization biases have a cumulative effect as the biased percepts of one generation influence the perceptual models that are passed on to the next. In this way, perceptual biases are a factor in regularizing the phonotactic structure of languages. All of this suggests that phonotactic repair may provide a window into some of the mechanisms that contribute to these central properties of human language. In this paper we examine the dynamic neural processes that support phonotactic repair.

Any account of phonotactic repair must address several basic facts about phonotactic competence. The first is that phonotactic constraints are bounded, but not entirely determined, by perceptual and articulatory demands. Sequences of speech sounds must be pronounceable and discriminable. A broad body of experimental and theoretical research has established a relationship between perceptual and articulatory constraints and patterns of preferred (less marked) phonological and phonotactic patterns (c.f. [8], [9]). However, differences between languages demonstrate that phonotactic patterns cannot be explained as a sole function of articulatory or perceptual constraints, since patterns that are legal in one language (e.g./sr/as in/srαzu/− Russian for “immediately”) are not permitted in others (e.g. */sr/is not a legal onset in English). Moreover, some phonotactic patterns are unattested even though they are readily produced and highly discriminable [10].

Our understanding of phonotactic repair must also address the productivity of phonotactic processes. In addition to showing a preference for sound patterns that are found in words they know [11], listeners show systematic preferences for some unattested patterns over others. For example, while several languages including English and Korean lack the onset clusters *bn*, *bd* and *lb*, speakers of these languages show a consistent pattern of preference: bn>bd>lb that is reflected in rates of perceptual repair [6], [12]. This suggests that listeners do not simply memorize a list of acceptable and unacceptable forms.

There are three broad accounts of the nature of phonotactic repair. Two focus on the nature of phonotactic knowledge, with one approach ascribing it to tacit knowledge of abstract rules, and another to tacit knowledge of the statistical properties of speech. A third account suggests that phonotactic repair is the result of the mapping dynamics that link speech input to stored representations of words.

Rule-driven or symbol manipulation accounts suggest that language learners discover abstract rules that describe relationships between potentially open sets of elements (e.g. speech sounds that share a common feature or characteristic) termed variables [13], [14], [15], [16]. In practice, repair would occur when a rule violation is detected, and would be constrained by the rules. Examples of abstract rules or constraints include the Sonority Sequencing Principle [17], which asserts that any consonant sequences at the beginning of a syllable must show a pattern of ascending sonority (airflow or loudness [18]), and the Obligatory Contour Principle [19], [20], [21],which bars structures with certain consecutive identical features. Both principles have the effect of maximizing the perceptual contrast between adjacent speech sounds, which may facilitate speech perception [22]. These principles capture broad patterns of phonotactic constraint both within and across languages. Rule-driven frameworks account for the systematicity of phonotactic patterning [23] and provide a natural explanation for the generalization of phonotactic principles to unattested forms [16].

However, the rule- and constraint-based literature on how phonotactic phenomena are represented remains unsettled and incomplete. One problem is that there are violations to phonotactic rules. For example, the *st-* onset in *stand* and *step* violates both the Sonority Sequencing Principle and the Obligatory Contour Principle. A number of attempts have been made to address these exceptions including proposing a separate set of constraints to govern *s-* consonant cluster onsets [24], or arguing that the *s-* in these clusters either falls outside of the syllable [25] or forms part of a complex segment [26]. Alternatively, phonetically-grounded phonological theory suggests such phenomena are better captured by a system of interacting violable constraints that favor phoneme sequences with perceptually robust feature cues [27]. It is unclear whether the unsettled aspects of these accounts represent the temporary limits of current understanding, or intrinsic limits of this approach to account for all available data.

Turning from representations to processing, behavioral data demonstrate human listeners, including young infants, are capable of learning and applying simple abstract rules governing the patterning of speech sounds. In one study, Marcus et al. [28] exposed infants to sequences of nonsense syllables with patterns of element repetition governed by simple algebraic rules (e.g. ABA or ABB). They found that infants subsequently showed longer listening times to sequences of different nonsense syllables that failed to reflect these rules. This finding is consistent with a larger artificial grammar learning literature that suggests that listeners are able to abstract more complex rules, including rules directly modeled on the syntax of natural human languages [29]. While this literature is primarily aimed at syntax, the elements that are used in this work typically consist of nonsense syllables and the rules that are learned might be considered to be broadly phonotactic.

Neural data provide some evidence in support of rule-driven phonotactic processes. BOLD imaging studies have implicated a number of brain structures in the learning and use of abstract rules related to perceptual categorization, the performance of motor sequences and language-like processing [29], [30], [31], [32]. However, it is unclear whether damage to any of these areas influences phonotactic competence. While some aphasics produce phonological paraphasias such as calling a spoon a *spool* or *spoom,* the speech errors they produce are overwhelmingly phonotactically well-formed [33]. In some instances, aphasic speech errors show a systematic bias for structures that are more common cross-linguistically. This has been interpreted by some as evidence for a change in the operation of phonological constraints [34]. In related work, Buchwald et al. [35] argue that clearly articulated epenthetic simplifications of cluster onsets in one aphasic subject suggest a phonological locus for some speech errors. However, these errors also produce phontactically viable structures. Both types of errors contrast with the agrammatic speech of some aphasics, which is both simplified, and syntactically ill-formed [36]. An alternative interpretation is that languages generally favor structures that are relatively easily to produce and accurately perceive [9], and that aphasics simplify their output due to reduced processing resources. In the case of discrete phonological epenthesis, it is unclear whether speech errors result from changes in phonotactic constraints or from a (possibly intentional) strategy for avoiding difficult articulatory sequences. Discriminating between these accounts is difficult in part because there are no available data bearing on the question of whether aphasic patients show the selective loss of the ability to evaluate phonotactic well-formedness or produce phonotactic perceptual repair.

Statistically driven accounts [37], [38] argue that listeners are sensitive to how often they encounter specific sequences of phonemes or phonetic/phonological features and that they show a perceptual and articulatory bias towards more common sequences. Within this framework, phonotactic repair could be the result of a frequency-weighted feedforward mapping that biases listeners towards higher frequency phonological interpretations of speech input. In this case, phonotactically illegal sequences, which are zero or near zero frequency events, would produce weak feedforward mappings that would be overwhelmed by mappings that produce more common phonotactic sequences. This type of frequency sensitivity is a central phenomenon in many areas of human and animal perception and learning including human language processing [39], [40].

The main difference between rule-based and statistical accounts has to do with the role of induction. To the degree that different languages observe different phonotactic patterns, induction must play a role in any rule-based account. This involves the induction of specific rules in classical generative phonology [14], or the induction of constraint ranking in optimality theory [21]. This perspective is often taken to imply the existence of a dissociable mechanism for learning and applying grammatical principles. In contrast, statistical mechanisms may rely on local frequency sensitivity that is built into the feedforward mapping between speech sounds on phonological representations without a role for global induction. As a result, there is no need for a dissociable induction mechanism, and no need to account for phonotactic phenomena (e.g. the viability of the English *st-* onset cluster) that resist systematic characterization.

Research into statistical accounts has primarily focused on understanding the degree to which statistical properties of the lexicon predict nonword acceptability judgments, and nonword repetition performance [37], [38], [41], [42]. This includes work showing that phonotactic distribution statistics can predict subjects’ preferences among non-attested onset clusters, and capture wide-ranging phenomena across a set of 33 tested languages including vowel harmony and stress patterning [41]. However, current models fall short in at least one respect. Berent et al. [43] have shown that the most effective current computational model, Hayes and Wilson’s maximum entropy model or Maxent [41], fails to predict human judgments about the well-formedness of Hebrew root structures containing nonnative phonemes. This type of generalization is documented in human listeners, and emerges naturally from rule-driven accounts of phonotactic competence [44].

The third approach attributes phonotactic competence to top-down lexical influences on speech processing. Like statistical approaches, this approach suggests that phonotactic constraints on perception are projected from the lexicon. However, the two have strikingly different functional processing architectures. Statistical models rely on modular mechanisms, while top-down mechanisms are by definition interactive. Quantitatively, they differ in the degree to which processing is influenced by the resemblance between input and specific lexical candidates. Thus a phoneme pattern with low-bigram frequencies and a small neighborhood might be disfavored by statistical analysis (e.g. *mouf or mouth*), but could benefit from lexical-feedback from a matching or highly similar attested word (e.g. *mouth*). In this way, top-down lexical influences on speech perception may facilitate the processing of both statistically favored and disfavored words. These top-down processes are hypothesized to contribute to the robustness of speech perception - a central challenge to our understanding of language processing given the lack of invariance in the mapping between speech sounds and phonemic categories [45]. The contrast between these two perspectives is the focus vigorous debate in the speech perception literature (cf. [46], [47], [48]).

The interactive activation TRACE model [49] provides an explicit model of how top-down lexical influences on speech perception might produce phonotactic repair. The TRACE model takes featural representations as input. These are linked to phonemic representations that are in turn linked to lexical representations. All connections between layers are excitatory and reciprocal, and nodes within the phonemic and lexical layers have inhibitory connections. The TRACE model produces phonotactic repair through top-down lexical influences on phonemic activation that are amplified through phoneme-to-phoneme competition. In one TRACE simulation, a segment that was ambiguous between/*l*/and/*r/*was presented in the context/*s_i*/, creating a possible legal interpretation (*sli*) and a potential illegal interpretation (*sri*). The/l/and/r/nodes initially showed similar activation, but over time the activation of the phonotactically legal/l/node became stronger and the activation of the illegal/r/node became weaker. The TRACE model does not learn, and so there is no mechanism that could support the discovery of either abstract rules or co-occurrence statistics. Instead, partial activation of words that begin with *sl-* (*sleek*, *sleep*) provided top-down activation of/l/. No words in the lexicon begin with sr-, and so there is no equivalent source of top-down activation for the/r/node. Because inhibition is proportional to activation in TRACE, this increased activation of the/l/node increasingly depressed activation of the/r/node. The implication of this result is that this type of phonotactic repair is an obligatory consequence of top-down lexical influences on speech perception.

There are two general challenges for the account. The first is the question of whether such a mechanism could account for listeners’ systematic preferences among unattested clusters. This remains an open question. It should also be noted that the notion of top-down lexical influence is not inconsistent with the possibility that bottom-up frequency sensitive mechanisms also contribute to human performance. Such capabilities could be built into a TRACE or TRACE-like interactive activation model such as TISK [50]. However, top-down and bottom-up mechanisms are dissociable and so should be considered separately.

Even if simulation could establish the computational adequacy of interactive activation, one would still be faced with the problem of determining whether listeners rely on interactive processes in speech perception. Standard behavioral and BOLD imaging techniques have fundamental inferential limitations that make this a difficult task [51]. The TRACE results argue that repair is an inevitable consequence of top-down lexical influences on speech perception. A wide range of behavioral and neural data demonstrate that lexical factors influence perceptual judgments about speech stimuli (c.f. [52], [53], [54], [55]). These results are consistent with the view that the lexicon directly influences speech perception. However, Norris et al. [56] offer alternative interpretations of many of these results, suggesting that lexical and prelexical representations may interact at a post-lexical decision phase rather than through the direct top-down processes suggested by the TRACE model. Behavioral experiments that rely on explicit perceptual judgments are inherently unable to discriminate between these alternatives because judgments are made after either top-down or bottom-up processes are completed [56], [57]. Standard BOLD activation analyses are similarly limited because feedforward and feedback models predict the same spatial pattern of activation [53]. It is similarly challenging to distinguish between putative lexical effects, and the potential effects of phonotactic frequency derived from the structure of the lexicon [46], [47], [48].

In order to distinguish between these accounts, it is necessary to disentangle the tightly convolved effects of rules, the lexicon, and the statistical distribution of the elements that comprise the lexicon. Natural language manipulations of any of these factors are inherently confounded with unintended manipulations of the other factors. Thus, an illegal or marked phonotactic cluster will also have low phonotactic probability and will have few lexical exemplars to support it. This makes behavioral testing difficult. Simulation approaches are equally problematic because any simulation is necessarily grounded by strong assumptions about phonological representation (e.g. which features to represented, or what units to count) that are often open to question. For these reasons, we have adopted a novel strategy that draws on differential predictions about the patterns of effective connectivity (non-correlational directed influence) between brain regions associated with acoustic-phonetic, lexical, and rule-governed processing shown by listeners during processing that leads to the perception of phonotactically legal versus illegal consonant clusters.

We used Granger analysis to evaluate effective connectivity. Granger analysis identifies patterns of directed causality (A → B, A ← B, and A ← →B) without the use of *a prioi* models. Granger analysis is based on the intuition that causes precede and uniquely predict their effects. We used a variant that relies on Kalman filter techniques to predict changes in localized activation that allows the analysis of large networks and does not require assumptions about the stationarity of brain activity [58]. We applied these analyses to magnetic resonance (MRI)- constrained source space reconstructions of whole head magnetoencephalography (MEG) and electroencephalography (EEG). These data are well-suited to Granger analysis because they provide the temporal resolution needed to perform event related time series analyses, and the spatial resolution and coverage needed to associate activity measures with specific anatomical structures over all cortical surfaces. To the extent that activation in individual brain regions can be associated with particular cognitive functions based on the functional imaging and neurological literatures, this approach can discriminate between top-down and bottom-up processes, and can be used to identify processing interactions predicted by cognitive theory [51], [53], [59], [60].

We used this technique to examine the categorization of word-initial fricatives. English prohibits syllables that begin with *sr-* and *shl-* (denoted as */sr/and */∫l/in standard linguistic notation), but allow words that begin with the sequences *sl-* or *shr-* (*sleep*, *shrine*). As noted earlier,/sC/and/∫C/present special challenges for rule-based accounts of phonotactic constraints. Nevertheless, the generalization that these/sr/and/∫l/are disallowed in English holds, with clear exceptions limited to loan words such as *schlep*. Previous behavioral work confirms that listeners show the same general pattern of repair for these disallowed clusters that they do for other disallowed English consonant clusters [3], [4], which suggests that they do not represent a special case.

We created a 5-step continuum between/s/and/∫/and presented each step in nonsense syllables where they were followed by either an *–r* or *–l* and then a vowel. During testing, participants heard a syllable and then 500 ms later were shown a visual probe (the lateralized text S and SH). They were asked to indicate by left-handed button press which consonant best matched the sound at the beginning of the syllable. Simultaneous MEG and EEG data were collected while participants completed the task. Anatomical MR data were collected in a separate testing session.

In each case we are concerned with the contrast between instances in which phonotactic constraints do and do not bias observed phoneme categorization. The rule-driven account predicts that phonotactic repair will produce increased influence by brain regions associated with rule application on brain regions associated with either acoustic-phonetic representation, or post-perceptual response selection. The statistical account predicts that lawful (phonotactic bias-consistent) sequences (/sl/or/∫r/) will produce stronger feedforward effects by acoustic-phonetic areas on brain regions associated with phonological or lexical representation. The lexical influence account predicts that trials that produce phonotactic bias consistent responses will show stronger top-down influences on acoustic-phonetic regions by regions implicated in lexical representation. These predictions are not exclusive, opening the possibility that phonotactic repair is driven by a combination of mechanisms.

## Methods

### Participants

Fourteen right-handed native speakers of American English with no discernible auditory processing deficits participated in the study. All subjects provided written informed consent following a protocol approved by the Partners Healthcare Institutional Review Board. Of these, one subject was dropped due to a magnetic artifact and three were dropped due to the absence of a strong reliable behavioral effect. The remaining 10 subjects (5 women) had a mean age of 28.6 years.

### Stimuli

The stimuli consisted of a 5-step [s] – [∫] continuum embedded at the onset of/_lV/and/_rV/contexts to create nonword CCV stimuli. The auditory stimuli were created from recordings of nonsense syllables spoken in isolation by a native speaker of American English. Items were digitally recorded with 16-bit sound at a sampling rate of 44.1 kHz in a quiet room. The fricative continuum was created through weighted spectral averaging of tokens of/s/and/∫/spoken in isolation and equated for duration at 80 ms using PRAAT (http://www.praat.org). An 11-step continuum was created and used in pilot behavioral testing. Based on the results of piloting this continuum was reduced to 5 steps (originally steps 0,3,5,7 and 10, labeled steps 1–5 in the scanning study). These fricatives were cross-spliced with tokens of the syllables/læ/,/ræ/,/lε/,/rε/,/l<$>\raster="rg1"<$>/,/r<$>\raster="rg1"<$>/,/l<$>\raster="rg2"<$>/and/r<$>\raster="rg2"<$>/that were digitally equated to a duration of 300 ms. The/læ/and/ræ/contexts were not used in the study conducted in the scanner. All stimuli were normalized for mean amplitude.

### Procedure

Magnetoencephalography (MEG) and electroencephalography (EEG) data were acquired simultaneously in a single testing session in a three-layer magnetically shielded room (Imedco, Hägendorf, Switzerland) while participants completed a delayed two-alternative forced choice phoneme categorization task. Each trial consisted of the presentation of a single auditory CCV token. This was followed by a 400 ms ISI and then the presentation of the lateralized visual response probes “S” and “SH”. The lateralization of these probes was randomized with “S” and “SH” appearing an equal number of times in the left and right side positions. Subjects were given two response keys and were instructed to press the key with their left hand that was on the same side of the keypad as the response prompt that corresponded to the initial speech sound they heard. Delayed randomized probes were used to eliminate anticipatory responding. Response probes appeared on screen for one second. Time between stimuli was drawn from a uniform distribution with a mean 400 ms, minimum 325 ms, maximum 475 ms. each stimulus was presented 30 times for a total of 900 trials that were broken down into 6 blocks. 180 additional filler trials distributed over the 6 blocks were also administered in which the actual fricatives were immediately followed by a vowel with no intervening consonant.

### MEG and MRI

MEG data were collected using a 306-channel whole head Neuromag Vectorview system (Elekta, Helsinki, Finland). The Vectorview system has 204 planar gradiometers and 102 magnetometers for collecting magnetic data, and incorporates a 70-electrode EEG cap with a nose reference for collecting electrical data as well as vertical and horizontal electro-oculograms (EOG). Both MEG and EEG data were recorded at a sampling rate of 1209.83 Hz after filtering between 0.1 and 400 Hz. For subjects 1–3 the sampling rate was 606.15 Hz and the filtering was between 0.1 to 200 Hz. These data were subsequently upsampled to conform to the protocol. Trials were rejected based on eye movement or blink artifacts detected by EOG (EOG >150 µV), or high magnetometer (>100,000 fT) or gradiometer (>300,000 fT/cm) values. The positions of all scalp electrode, anatomical landmarks including the nasion and two auricular landmarks, and four head-position indicator coils were measured using a FastTrack 3D digitizer (Polhemus, Colchester, VT). During testing, the head position within the MEG sensor array was measured at the beginning of each of the six blocks and at the end of the testing session.

High-resolution 3D T1-weighted structural images (head-only) were acquired in a second testing session using a 1.5 T Avanto 32 channel “TIM” system using an MPRAGE sequence (TR = 2730 ms, T1 = 1000 ms, TE = 3.31 ms, flip angle = 7°, FOV = 256 mm, slice thickness = 1.33 mm).

### Region of Interest Identification

We identified regions of interest (ROIs) automatically using an algorithm that relied on the mean strength and similarity of activation time series recorded at all vertices over the cortical surface. Subject activity maps were morphed into an average brain and then averaged together for the selection of study-wide regions for each condition. Based on these activity maps, vertices were chosen to represent regions and expanded outward to form contiguous regions of interest. These regions were mapped back onto the subject brains and representative points were determined by regionally maximal cortical activity based on the previous MNE maps.

The algorithm that defined regions of interest operated in three stages. In the first stage, vertices with mean activation over the 95^th^ percentile in the 100 ms to 400 ms post-stimulus onset time window were selected to be candidate centroids for regions of interest. Vertices within 5 mm of local maxima were excluded from candidacy. This yielded approximately 50 to 150 candidate centroids. The second stage iterated through each candidate centroid, in order of highest activity to lowest, and formed a region around it based on similarity. Similarity was determined by taking the negation of the Euclidean norm of the difference between the brain activity waves after normalization (demeaning and division by the standard error). Spatially contiguous vertices with brain activity waves of similarity within 0.5 standard deviations of the centroid’s activation function were included with the centroid to define its ROI. This defined regions of homogenous activation from which representative vertices could be identified in each subject’s data. The third stage, embedded in each iteration of the second stage, removed all candidate centroids not yet formed into ROIs with activation functions within 0.9 standard deviations of previously chosen centroid functions. This step is required to meet the assumption of Granger analysis that all time series carry unique information.

### Kalman Filter-Based Granger Analysis

Inter-regional influence was computed using Granger causality [61] analyses based on Kalman filter techniques [58]. Average brain activity waves from each subject at each of the representative points of the conditional ROIs were submitted to a multi-trial Kalman filter. Kalman filters address the non-stationarity of neural activity by using an adaptive, automatically scaled wave transformation. The original Kalman model with all regions of interest was computed, and then one counter model was generated for each ROI without the presence of the ROI. The 5 samples prior to each frame were used to determine a basis for the next frame in each Kalman model iteration. This model order was identified heuristically after Akaike Information Criteria and Bayesian Information Criteria analyses failed to identify a single optimal prediction lag. It took about 50 ms for the model to converge; the model was computed over time from 0 ms to 600 ms.

Granger-Causality was inferred using the ratio in standard prediction errors. For each pair of ROIs, A and B, if a region B’s standard prediction error is greater in the model without region A than in the model with region A, it is inferred that the presence of A in the model can be used to predict region B’s activity, and therefore region A Granger-causes region B. The logarithm of the ratio of the standard prediction error in the full model versus the omitted model was used to compute the Granger Causality Index (GCI) at each point in time [58].

The significance of Granger Causality Indices was determined using a bootstrapping method [58] to form a GCI threshold. Alternative models were generated and tested through the Kalman-Granger procedure. These models used data reconstructed from the Kalman matrices of the initial model, excluding inter-ROI projections from one ROI at a time and randomizing the residuals. Two thousand models were generated and an independent distribution of GCIs was established for each directed ROI-ROI interaction for each time point. These distributions were used to assign probability estimates to each computed GCI value. Comparisons of the relative strength of Granger influence between conditions were made using a permutation test. The p-value for rejecting the null hypothesis that the number of p-values below 0.05 is the same for the same directed link in two conditions (based on the same ROIs) is p = 0.05.

## Results

**Figure 1:**
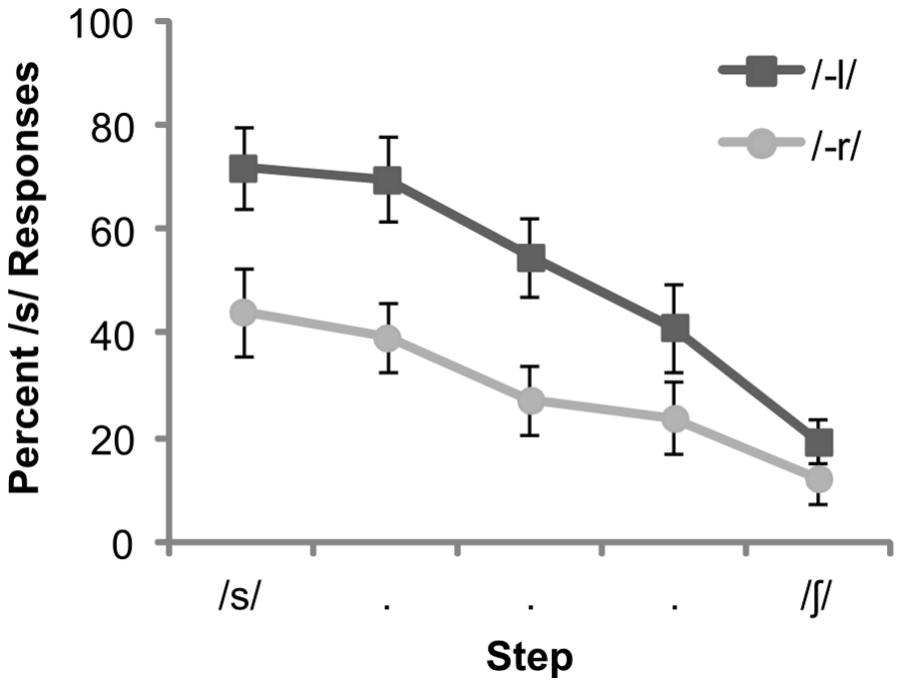
Behavioral results for the fricative categorization task for fricatives (F) presented in the context of/Fl−/versus/Fr−/clusters (error bars = ±1 SE).

Behavioral results showed a strong phonotactic influence on categorization (Fig. 1). Analyses were based on the percentage of “S” responses. There was a main effect of context [F(1,9) = 894.59, P<0.001], with subjects favoring the phonotactic bias consistent S interpretation in the *–l* context, and SH interpretation in the *–r* context. In addition, there was a main effect of continuum step [F(1,4) = 394.24, P<0.001, with subjects showing a greater tendency to classify tokens at the/s/end of the continuum as S]. There were no significant interactions (p>0.05).

Analyses of effective connectivity focused on the interval between 200 and 400 ms after stimulus onset. We selected this interval based on evidence that listeners show electrophysiological sensitivity to native phonotactic violations in this time period [62], [63] We used Granger analysis techniques to examine patterns of effective connectivity in this time period in trials involving acoustically unambiguous tokens (steps 1 and 5). We chose these tokens to minimize the influence of dynamics specifically related to perceptual ambiguity (e.g. the Ganong effect) and to isolate dynamics more directly attributable to phonotactic processes. The observations were broken down into two conditions based on participants’ categorization of the fricatives. One group included trials in which categorization yielded a legal or bias consistent cluster (e.g. labeling a fricative “S” in the/l/context or “SH” in the/r/context), and the other consisted of trials in which categorization produced an illegal or bias inconsistent cluster (e.g. labeling a fricative “S” in the −/r/context or “SH” in the −/l/context). Source and sensor space activation patterns for these conditions are shown in Figures S1–S3. Regions of interest (ROIs) were identified automatically using an algorithm that identified clusters of vertices associated with activation peaks showing common temporal activation patterns, and then compared the time course of all clusters to eliminate ROIs that provided redundant information. This analysis was based on all trials so that we could directly compare the strength of interactions between a common set of ROIs supporting phonotactically consistent versus inconsistent responses.

**Figure 2:**
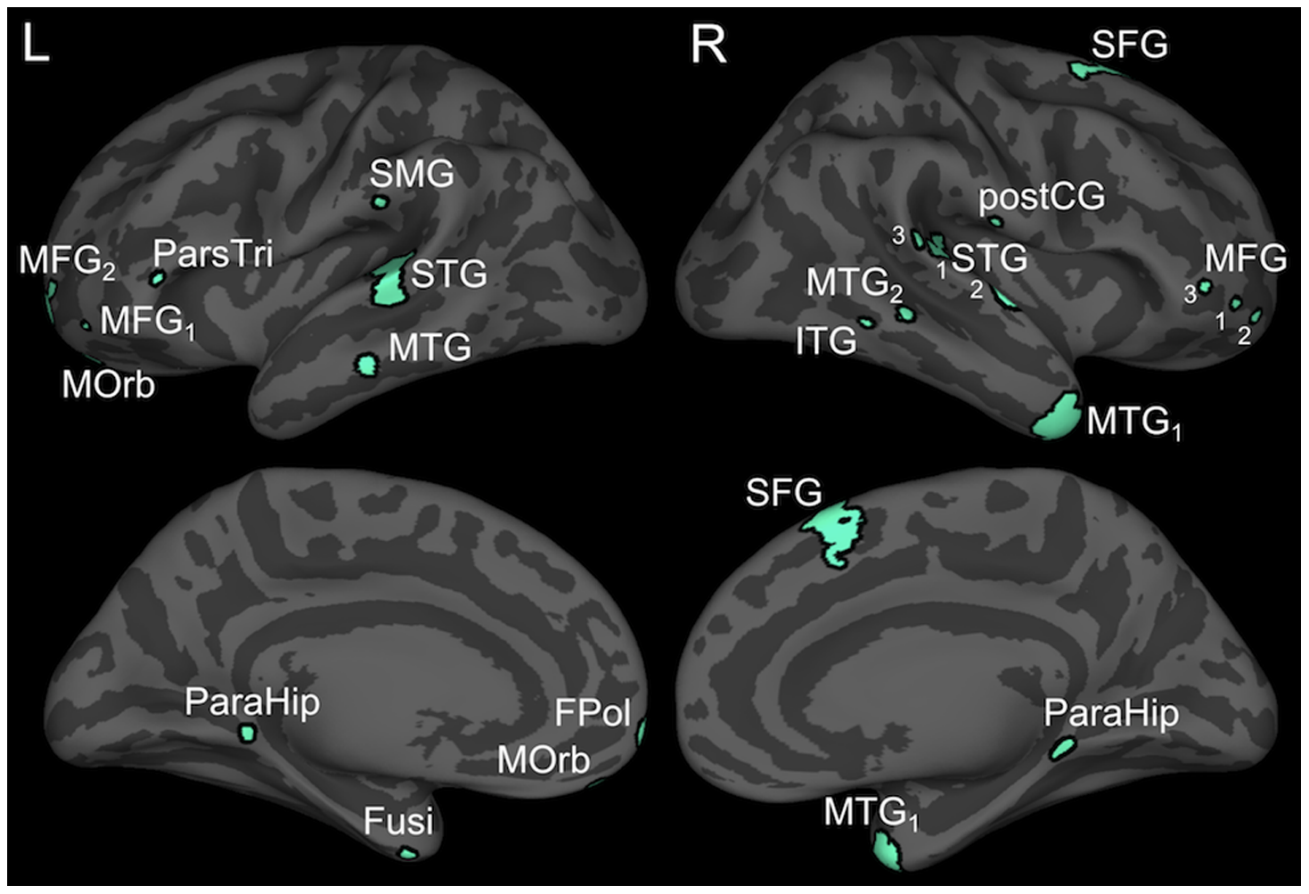
Regions of interest visualized over an inflated cortical surface.

We identified 22 ROIs (Table 1 and Figure 2). Because nearly all ROIs influenced each other to some extent in both conditions, we limited our analyses to interactions directly implicated in the three accounts of phonotactic competence. Critical analyses focused on interactions involving the left posterior STG, because convergent results from BOLD imaging and source localized electrophysiological studies show that this region is sensitive to phonotactic violations [63], [64], and is implicated in acoustic-phonetic processing [65]. While these earlier results show that this region plays a central role in the processing of phonotactic violations, they are silent on the question of the nature of this role. By probing how pSTG interacts with other regions, we hope to clarify its role. If phonotactic repair involves the modification of perceptual representations, Granger analysis should reveal stronger top-down influences on left pSTG activation in trials that produce phonotactically consistent responses. A rule-driven account would predict that this would come from an ROI associated with rule application, while the lexical influence explanation would predict influence by a region associated with lexical representation.

**Table 1:** Regions of interest (ROIs) used in all Granger analyses.

Label	Location	MNI Coordinates (X, Y, Z)		
**Left**				
FPol	Frontal pole	−13.93	67.55	−6.39
Fusi	Fusiform gyrus	−27.56	1.6	−45.74
MFG_1_	Middle frontal gyrus	−34.22	58.2	−7.74
MFG_2_	′′′′	−22.58	62.5	−0.06
MOrb	Medial orbital gyrus	−7.57	50.46	−27.18
MTG	Middle temporal gyrus	−60.33	−23.26	−21.05
ParaHip	Parahippocampus	−11.91	−40.21	−6.88
ParsTri	Pars triangularis	−53.81	30.91	1.35
SMG	Supramarginal gyrus	−65.01	−28.08	20.8
STG	Superior temporal gyrus	−64.59	−30.13	−1.67
**Right**				
ITG	Inferior temporal gyrus	56.24	−48.3	−10.19
MFG_1_	Middle frontal gyrus	39.01	54.51	−2.49
MFG_2_	′′′′	30.04	59.09	−6.6
MFG_3_	′′′′	42.53	42.29	0.79
MTG_1_	Middle temporal gyrus	44.3	10.62	−41.83
MTG_2_	′′′′	66.25	−35.59	−3.86
ParaHip	Parahippocampus	15.47	−35.47	−9.12
SFG	Superior frontal gyrus	10.87	24.13	60.38
STG_1_	Superior temporal gyrus	57.56	−28.51	9.75
STG_2_	′′′′	58.45	−6.11	2.88
STG_3_	′′′′	66.36	−30.48	14.03
postCG	Postcentral gyrus	63	−14.16	15.38

MNI coordinates refer to the voxel showing the highest mean MNE activation over the 200–400 ms post-stimulus onset interval for each ROI.

**Figure 3:**
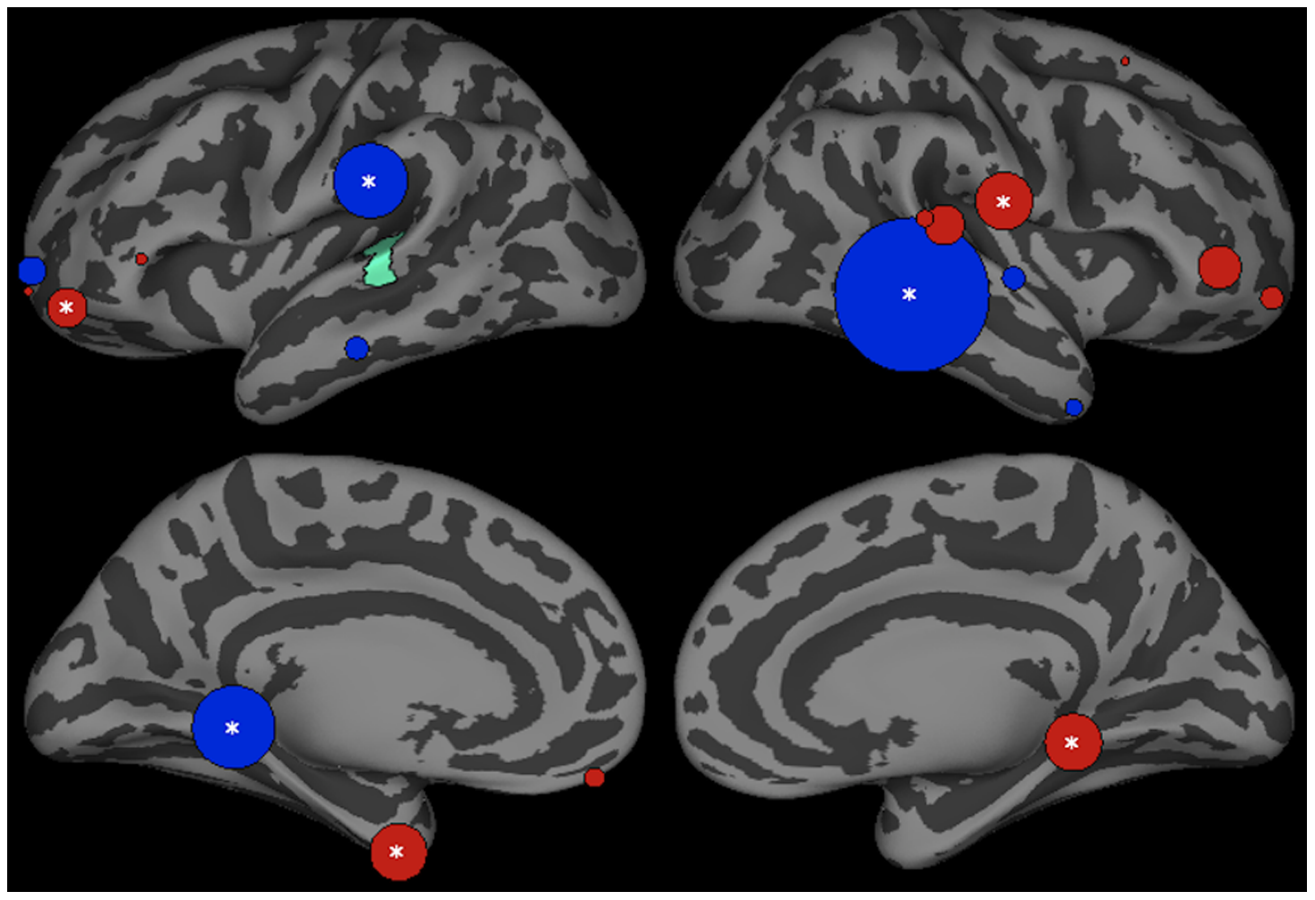
Top-down influences on left pSTG (green) activation in the interval between 200–400 ms after stimulus onset in trials producing phonotactic bias consistent (legal) and inconsistent (illegal) phoneme categorization. Bubble size indexes the relative strength of Granger influences (number of time points that show GCI values with p<0.05) of ROIs on left pSTG activation. Regions showing stronger bias consistent>bias inconsistent trials are shown in blue, and bias inconsistent>consistent are shown in red.

Influences on left pSTG activation are shown in Figure 3. Three ROIs, the left parahippocampal region (p<0.001), left supramarginal gyrus (SMG) (p<0.01) and right middle temporal gyrus region 2 (MTG-2) (p<0.001) had significantly stronger influences on pSTG activation in trials that produced phonotactic bias consistent versus inconsistent phoneme categorization responses. Consistent with the top-down lexical influence hypothesis, both the left SMG and bilateral posterior MTG are hypothesized to store word-form representations [65], [66]. Previous work using very similar methods has shown that SMG and MTG influence on the left posterior STG contribute to lexical effects in the interpretation of speech sounds [53], [59]. The left parahippocampal region has been shown to play a role in the acquisition of novel rules, but this role seems to disappear after acquisition [29], [31]. Stronger influences were found in trials that produced phonotactically illegal responses in the left fusiform region (p<0.01), a left rostral middle frontal gyrus region (rMFG1) (p<0.05), the right parahippocampal region (p<0.05) and right posterior central gyrus (postCG) (p<0.05).

**Figure 4:**
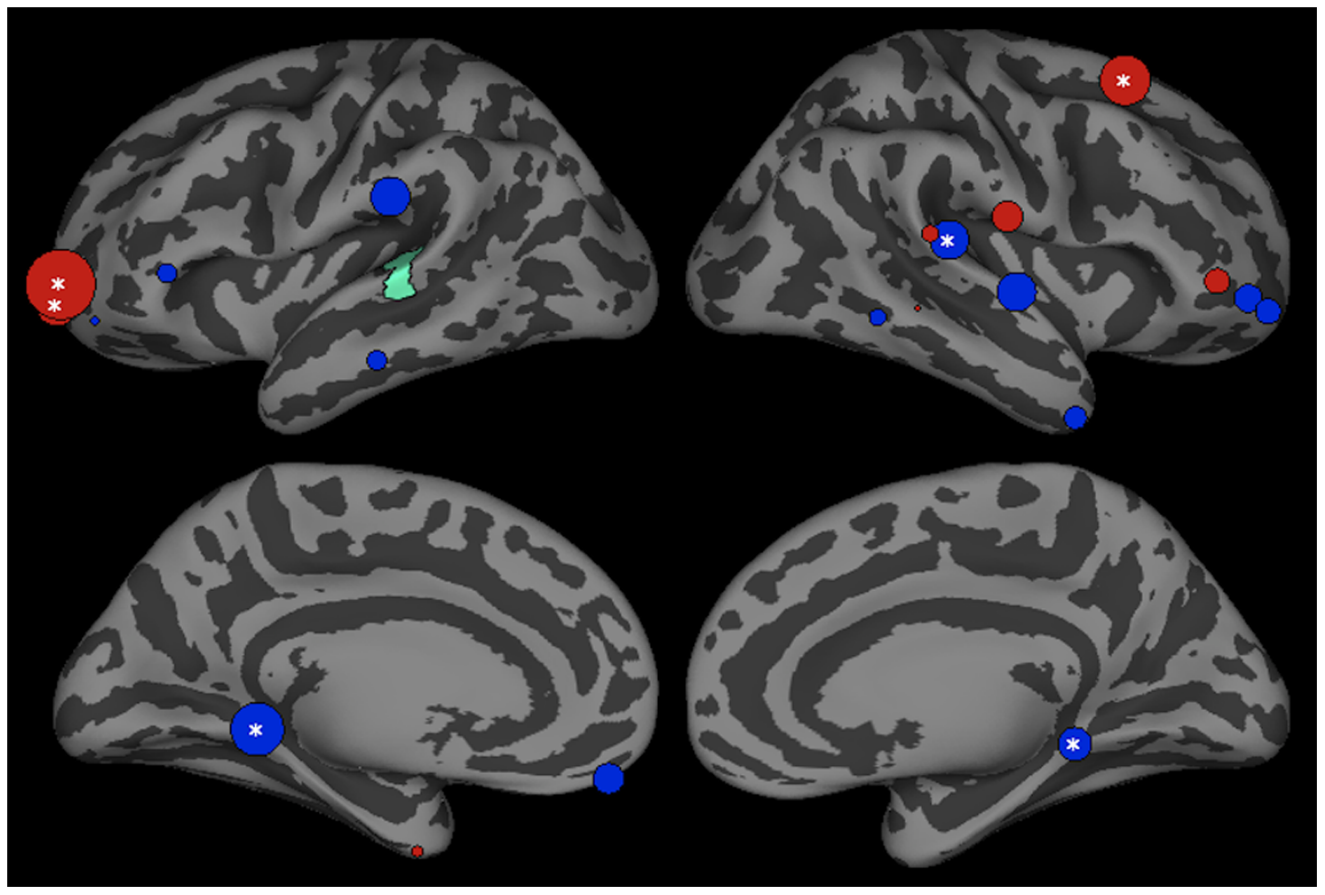
Bottom-up influences by left posterior STG (green) between 200–400 ms after stimulus onset in trials producing phonotactic bias consistent (legal) and inconsistent (illegal) phoneme categorization. Bubble size indexes the relative strength of Granger influences (number of time points that show GCI values with p<0.05) of ROIs on left pSTG activation. Regions showing stronger bias consistent>bias inconsistent trials are shown in blue, and bias inconsistent>consistent are shown in red.

The statistical account predicts that feedforward mapping from the left pSTG to regions associated with higher phonological representation should be facilitated for phonotactic bias consistent sequences because they occur more often than illegal sequences. Feedforward influences of the left STG on other brain regions are shown in Figure 4. Stronger influences are shown in trials that produce phonotactic bias consistent responses in the left (p<0.01) and right parahippocampal regions (p<0.05), regions associated primarily learning and episodic memory representation [67]. In trials that produce phonotactic bias inconsistent responses, the left STG had a greater influence on the left frontal pole (FP) (p<0.01), left rMFG (p<0.001) and right superior frontal gyrus (SFG) (p<0.001) – regions implicated in cognitive control and response selection [68], [69].

**Figure 5:**
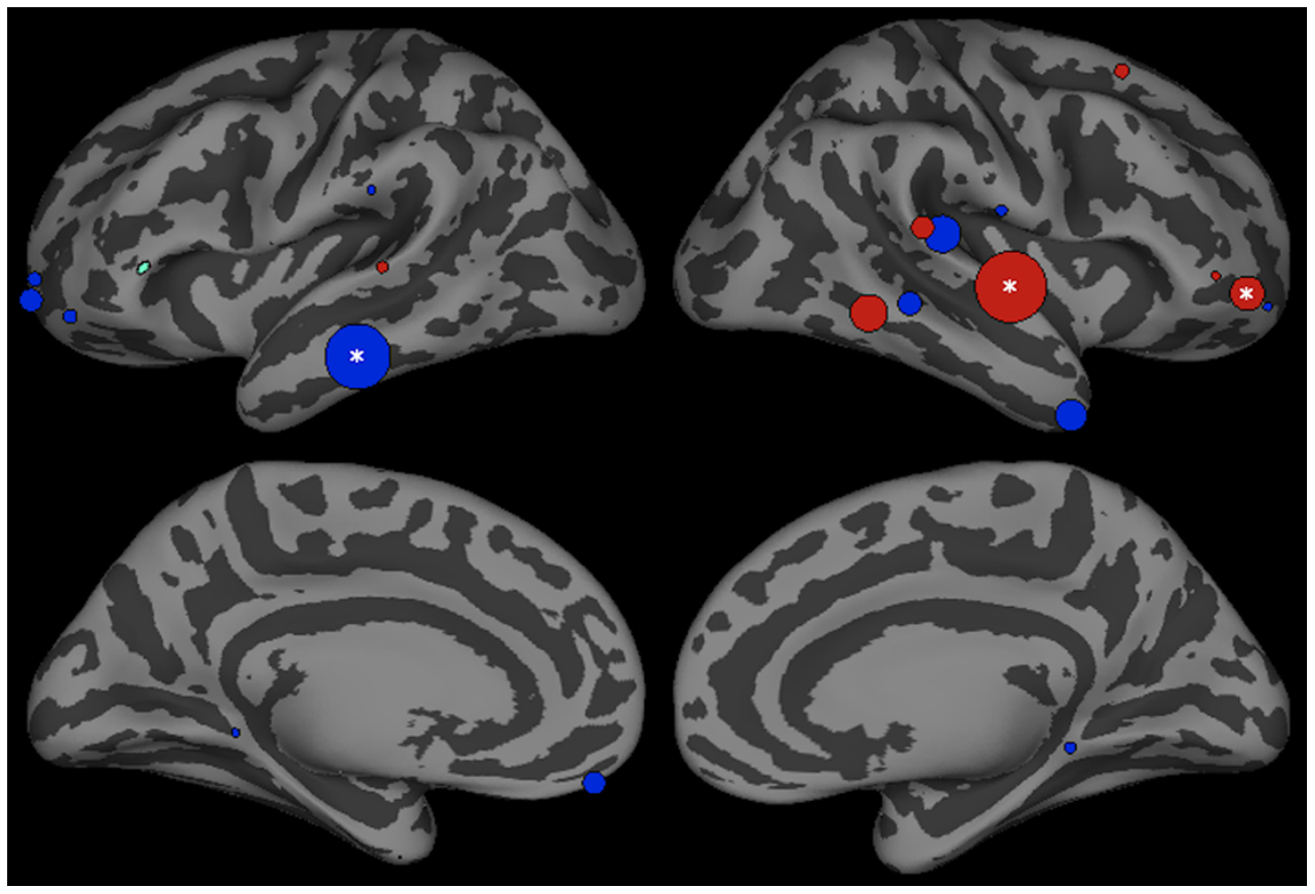
Influences of left pars triangularis (green) between 200–400 ms after stimulus onset in trials producing phonotactic bias consistent (legal) and inconsistent (illegal) phoneme categorization. Bubble size indexes the relative strength of Granger influences (number of time points that show GCI values with p<0.05) of ROIs on pSTG activation. Regions showing stronger bias consistent>bias inconsistent trials are shown in blue, and bias inconsistent>consistent are shown in red.

An additional set of analyses examined the influences of the left pars triangularis (PT) on other ROIs (Figure 5). While the parahippocampal regions and FP have been shown to play a role in the learning of novel rules, the left PT is the only ROI in our set that has been implicated in the execution of rule-driven (as opposed to similarity-driven) judgments in the BOLD imaging literature [29], [30], [31]. A feedforward variant of the rule-driven account would predict that PT activation could have downstream effects on activation in other regions. The left PT showed stronger influence on the left MTG (p<0.001) in trials that produce legal phoneme categorization and stronger influence on the right STG-2 (p<0.001) and rMFG1 (0.05) ROIs in trials that produced illegal categorization.

## Discussion

This study tested neurodynamic predictions of three explanations of phonotactic biases on speech categorization. Our analyses revealed a complex pattern of interaction between brain regions, which suggests that none of these accounts provides a full description of the processes that support performance on the task. However, focused analyses give clear support for the predictions of the lexical influence account, but fail to strongly support the predictions of the rule-based on frequency accounts.

The lexical influence account predicts that brain regions that represent wordforms should drive activation more strongly in the left pSTG in trials that produce legal versus illegal phoneme categorization. Two such regions are the left SMG and right MTG [65], [66]. These roles are developed in detail in the dual lexicon model [66], which argues that the left SMG acts as a lexical interface between acoustic-phonetic and articulatory representation, and that bilateral MTG is an interface between acoustic-phonetic and semantic/syntactic representation. This framework is supported by functional imaging results show that activation in both regions is modulated by whole word properties including word frequency, and the phonological similarity of a word to other words [33], [70], [71], [72]. It is also supported by aphasiological reports that damage to the middle temporal gyrus leads to deficits in lexico-semantic processing and the production of lexical speech errors known as semantic paraphasias such as calling a spoon a *knife* [73], [74]. Similarly, damage to the left supramarginal gyrus is associated with deficits in lexico-phonological processing and the production of phonological paraphasias such as calling a spoon a *spool* or *spoom* [74].

These results parallel those of previous effective connectivity studies that show a relationship between increased influence by the SMG and MTG on STG activation and behavioral evidence for lexical influences on speech perception [53], [59]. This dynamic is hypothesized to support the robustness of speech perception in the face of variable or degraded speech input. The present results differ from previous results in two ways. First, unlike previous top-down influences on speech perception, the current results demonstrate that this dynamic extends to the perception of nonword stimuli, as predicted by interactive activation models of speech perception. In the TRACE model simulations, this is the result of top-down influences from words that overlap with the nonword stimuli [49]. This mechanism is consistent with behavioral evidence that the onset of a word produces parallel activation of a cohort of words that share the same beginning [75]. The notion that lexical representations can influence nonword perception is further supported by evidence for a relationship between statistical patterns found in the lexicon and performance on nonword acceptability judgments, reaction time in word/non-word judgment and non-word repetition accuracy [76], [77] as well as findings that these effects covary with vocabulary size in children [78].

Unlike behavioral and neural work exploring lexical influences on speech perception [52], [53], [79], [80], [81], the current results also suggest that lexical factors influence the perception of acoustically unambiguous speech sounds. Several studies have shown that listeners show behavioral evidence of phonotactic influences on the categorization of unambiguous speech sounds [3], [4]. This difference might suggest a dissociation between phonotactic and lexical influences on speech perception. Alternatively, it may be attributed to the fact that non-word alternatives in a typical lexical influence study (e.g. KIFT or GISS) may receive some lexical support from candidates that overlap at the onset (KISS, KICK, GIFT, GILL), while phonotactically ill-formed items (e.g. */sra/) receive no such support.

Evidence for a role of rule-driven processes in phonotactic repair was less compelling. Two of the ROIs identified in this study, the left parahippocampal region and left pars triangularis, have been shown to play a role in the learning and application of artificial grammars in BOLD imaging studies. The left parahippocampal regions involvement is particularly significant because this region produced significantly stronger influence on left pSTG activation in trials that produced phonotactically legal responses. Several studies have shown increased left hippocampal activation during the acquisition of language-like artificial grammars [30], [31], [82]. This activity may appear as parahippocampal activation in MEG reconstructions of cortical activity. In these studies, activation is found in a more anterior region than we found. This activation is associated primarily with novelty and is found in conditions that encourage categorization based on perceptual similarity rather than the application of explicit rules. All of these studies showed that this activation is markedly reduced over the course of learning. Opitz and Friederici [82] note that this reduction accompanied by an increase in left ventral premotor cortex activation that they interpret as a shift from similarity-based learning to rule-based abstraction. Because phonotactic rules were presumably well established in our subjects, it is unlikely that parahippocampal activation reflects rule acquisition. A more likely interpretation is that this activation is related to this region’s role in episodic memory encoding or retrieval [83].

The left pars triangularis may be more closely associated with rule-based processing. A number of studies have identified frontal regions that include the pars triangularis (identified variously as Broca’s area, the left inferior frontal gyrus or left prefrontal cortex that include the pars triangularis) as the substrate of both natural and artificial grammar application [c.f. 29,30,31,82,84,85]. It may be relevant that these results are based on studies concerned with syntactic or morphological, but not phonotactic rules. The one current BOLD imaging study of phonotactic processing showed increased activation associated with phonotactic repair in the left STG and SMG, but not in any frontal region [64]. In our study, the left pars triangularis did not show differential influence on left STG activation as a function of the legality of phoneme classification.

The left PT did show stronger influence on left MTG activation in trials that produced phonotactically legal responses. The finding that interaction between these regions is strongly implicated in lawful syntactic and morphological processes [86], [87], [88], [89] might suggest that this supports rule-driven processing. There are several problems with this interpretation. The first is the lack of strong evidence linking the PT damage to phonotactic deficits, or PT activation to phonotactic repair in BOLD imaging. This suggests that the PT’s role in phonotactic processing is non-obligatory. Evidence that the syntactic deficits may occur without PT damage [90] and that syntactic processing may occur without increased activation of the region [91] suggests an alternative characterization of its role in syntactic processing with implications for phonotactic processing. Thompson-Schill et al. [92] argue that the left ventrolateral prefrontal activation found across many cognitive tasks may be interpreted as a domain general role in cognitive control and selection. In the context of syntactic processing, this may take the form of selection related to the role a word plays in competing parses of an ambiguous sentence [87]. In the context of a non-syntactic phoneme categorization task this interaction may reflect the strategic selection of words that share a legal onset cluster to facilitate lexical influences on phonetic processing. In this way, PT influences could enhance lexically-driven phonotactic effects.

The predictions of the frequency account are not strongly supported by the current results. Higher frequency (phonotactically legal) responses were not associated with stronger feedforward influences by the superior temporal gyrus on any brain regions associated with higher, explicitly linguistic representations. The only regions that showed stronger feedforward influence leading to a higher frequency categorization were the bilateral parahippocampal regions. These regions are primarily associated with contextualization of episodic memory [83]. It is unclear why this STG-to-parahippocampal regions interaction would be modulated by phonotactic frequency or legality. Despite the lack of positive evidence, our results do not preclude a role for frequency sensitivity in early bottom-up superior temporal processing prior to interactions with higher linguistic areas. Behavioral evidence suggests that lexical neighborhood size and phonotactic frequency make simultaneous, independent contributions to judgments of wordlikeness [93], albeit with stronger contributions by lexical factors.

It is unclear how broadly these results generalize to other phonotactic processes. In this study the phonotactic violations involved a sonority profile (stop-liquid) that is broadly attested in English. In this case, attestation involved the presence of lexical candidates that supporting legal clusters (/sl−/,/∫r−/), but not the illegal clusters (*/sr−/, */∫l−/). While the linguistic analysis of s- initial clusters in English is unresolved, there is reason to suspect that the unattested/sr−/and/∫l−/clusters represent relatively weak phonotactic violations. This is important given the finding [6] that listeners show relatively weak repair of weak violations. Moreover,/s/and/∫/differ only in place of articulation - a contrast neutralized in some American English dialects whose loss listeners readily learn to accommodate [94]. These observations raise the possibility that the stimuli used in this study may both minimize the potential for rule-governed repair, and maximize the potential for lexically-mediated repair.

Another possibility is that phonotactic repair generally depends on the existence of lexically attested sequences that are perceptually confusable with unattested consonant sequences. Top-down lexical influences on phoneme categorization are dependent on similarity between the input stimulus and lexical models [95]. Consider the case of languages such as Japanese that do not allow complex clusters. Evidence from perceptual experiments [5] shows that speakers of these languages rely on vowel epenthesis rather than consonant category shifts to repair illicit consonant clusters. The primary epenthetic vowel in Japanese is [u]. Like the English epenthetic vowel schwa, [u] is the least sonorant vowel in its vowel system, and may be devoiced in some contexts [96], [97]. Lexical contexts with stronger vowels (e.g. *mikado* “emperor”) do not drive phonotactic repair of illegal clusters (e.g. the nonword *mikdo*) [98]. Furthermore, Japanese listeners fail to perceive epenthetic vowels in contexts where Japanese phonology requires vowels other than [u] [99]. These findings are problematic for a rule-governed account of phonotactic repair, but are consistent with the idea that repair is produced by top-down lexical influence from perceptually weak, and thus confusable sequences. Within the influence framework, listeners would repair sequences such as/tm−/that have no perceptually similar attested cluster patterns, with epenthetic schwa based on similarity to words such as *tomato* that may contain the reduced sequence/t?m−/. Online lexical influences on speech perception provide a plausible account of these results, but one that remains to be tested experimentally.

Lexical influences on speech perception fail to provide an obvious plausible account of other results. The most notable are those relating to the role of phonotactic constraints on the patterning of identical consonants in stems in Semitic languages. Hebrew, for example allows identical elements at the right edge of a stem (e.g. *xgg* in *xagag*, “he celebrated”), but not at the left edge (* xxg). In the case of attested forms, the wordlikeness of nonwords may be explained by application of either rule-based ([e.g. [20]) or similarity-based (e.g. [100]) models. When presented with nonword stimuli, Hebrew-speaking listeners show sensitivity to this constraint in lexical decisions involving stimuli with non-native features [44]. These results are consistent with the predictions of a rule-based model, but are not adequately modeled by the statistically-driven Maxent model [43]. While the feedforward, statistically driven dynamics that produce behavior in the Maxent model are fundamentally different from those supposed by a top-down lexical mechanism, these results suggest a likely limitation of any mechanism that draws on lexical similarity.

## Conclusions

In summary, our results suggest that top-down lexical influences on acoustic-phonetic processing are one of the drivers of phonotactic repair. This suggests that lexical influences on speech perception contribute to the systematicity and generativity associated with phonotactic processes. It is not clear how broadly these mechanisms apply, even within the restricted domain of phonotactic processing. Nevertheless, this work demonstrates both the viability of non-rule-based mechanisms for explaining aspects of lawful behavior, and the potential of effective connectivity analyses as a new tool to explore the mechanisms that produce such behavior.

## Supporting Information

Figure S1 **Evoked cortical activity over all MEG sensors for the period of −100 to 800 ms timelocked to the onset of auditory stimulus presentation for phonotactic bias consistent (red curves) and inconsistent (blue curves) trials.**
(PNG)

Figure S2 **Mean source space MNE activation between 200–400 ms for trials producing phonotactic bias consistent (legal) phoneme categorization.**
(PNG)

Figure S3 **Mean source space MNE activation between 200–400 ms for trials producing phonotactic bias inconsistent (illegal) phoneme categorization.**
(PNG)
