# Supplementary figures and images for: Rules from Words: A Dynamic Neural Basis for a Lawful Linguistic Process
Source: PLoS One. 2014 Jan 21;9(1):e86212. doi: 10.1371/journal.pone.0086212 (PMC3897659; doi:10.1371/journal.pone.0086212)

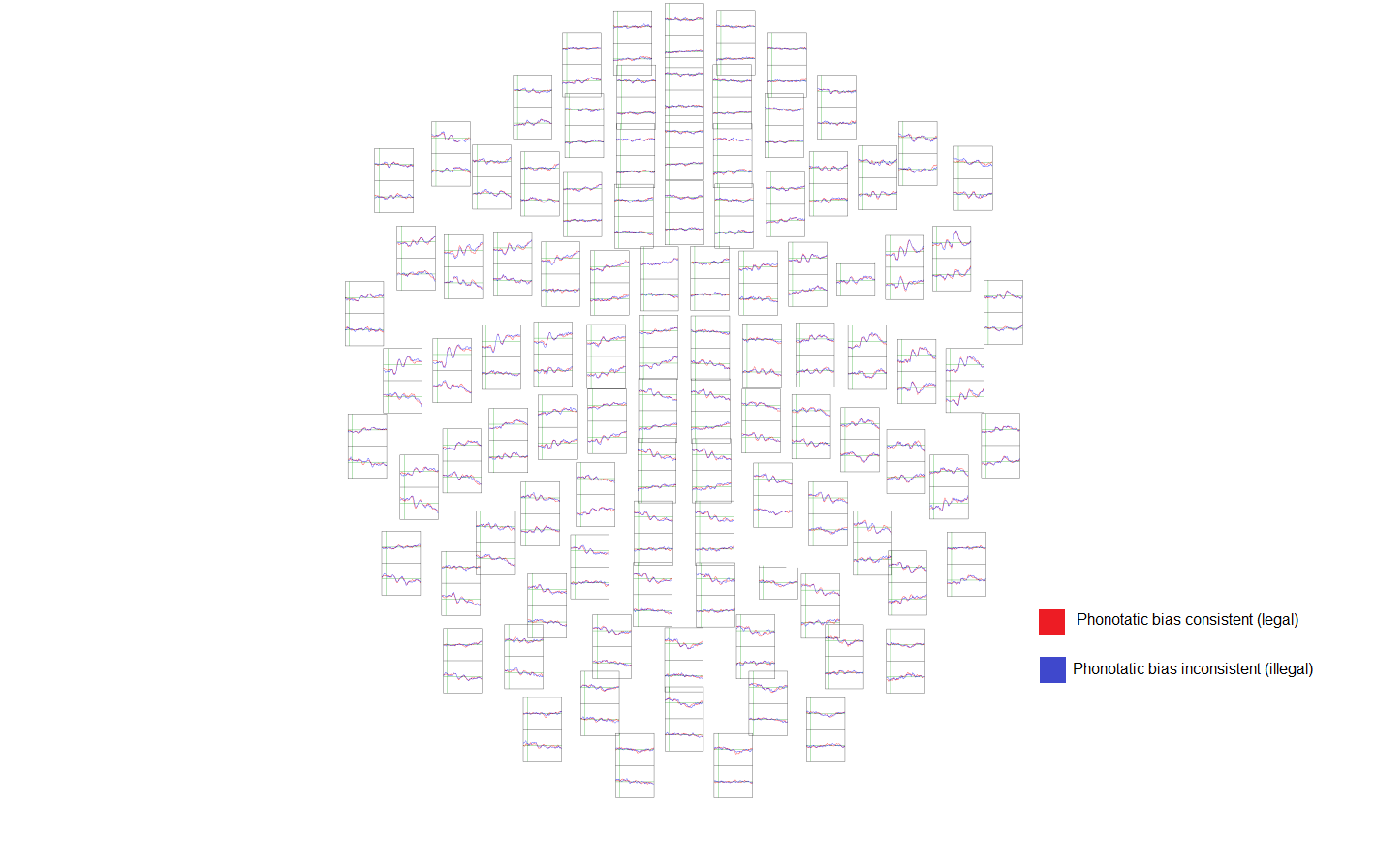

Supplement: Figure S1 — Evoked cortical activity over all MEG sensors for the period of −100 to 800 ms timelocked to the onset of auditory stimulus presentation for phonotactic bias consistent (red curves) and inconsistent (blue curves) trials. (PNG) [file pone.0086212.s001.png]

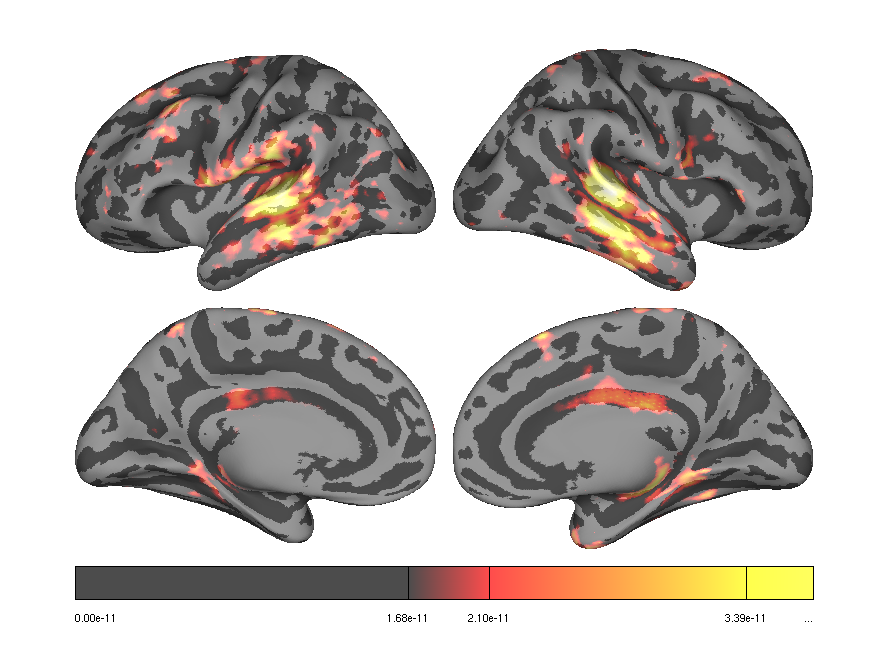

Supplement: Figure S2 — Mean source space MNE activation between 200–400 ms for trials producing phonotactic bias consistent (legal) phoneme categorization. (PNG) [file pone.0086212.s002.png]

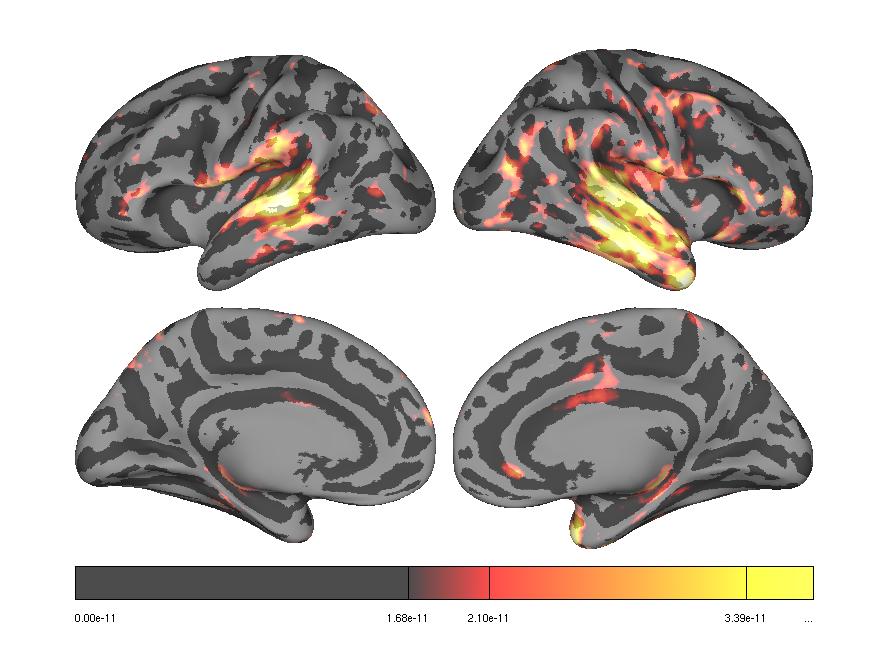

Supplement: Figure S3 — Mean source space MNE activation between 200–400 ms for trials producing phonotactic bias inconsistent (illegal) phoneme categorization. (PNG) [file pone.0086212.s003.png]
